# A method to advance adolescent sexual health research: Automated algorithm finds sexual history documentation

**DOI:** 10.3389/fdgth.2022.836733

**Published:** 2022-07-22

**Authors:** Caryn Robertson, Gargi Mukherjee, Holly Gooding, Swaminathan Kandaswamy, Evan Orenstein

**Affiliations:** ^1^Department of Pediatrics, Emory University, Atlanta, GA, United States; ^2^Children's Healthcare of Atlanta, Atlanta, GA, United States; ^3^Grady Memorial Hospital, Atlanta, GA, United States

**Keywords:** adolescent, natural language processing (computer science), sexual health, free text, sexually transmitted infections, regular expression (regex)

## Abstract

**Background:**

We aimed to develop and validate a rule-based Natural Language Processing (NLP) algorithm to detect sexual history documentation and its five key components [partners, practices, past history of sexually transmitted infections (STIs), protection from STIs, and prevention of pregnancy] among adolescent encounters in the pediatric emergency and inpatient settings.

**Methods:**

We iteratively designed a NLP algorithm using pediatric emergency department (ED) provider notes from adolescent ED visits with specific abdominal or genitourinary (GU) chief complaints. The algorithm is composed of regular expressions identifying commonly used phrases in sexual history documentation. We validated this algorithm with inpatient admission notes for adolescents. We calculated the sensitivity, specificity, negative predictive value, positive predictive value, and F1 score of the tool in each environment using manual chart review as the gold standard.

**Results:**

In the ED test cohort with abdominal or GU complaints, 97/179 (54%) provider notes had a sexual history documented, and the NLP algorithm correctly classified each note. In the inpatient validation cohort, 97/321 (30%) admission notes included a sexual history, and the NLP algorithm had 100% sensitivity and 98.2% specificity. The algorithm demonstrated >97% sensitivity and specificity in both settings for detection of elements of a high quality sexual history including protection used and contraception. Type of sexual practice and STI testing offered were also detected with >97% sensitivity and specificity in the ED test cohort with slightly lower performance in the inpatient validation cohort.

**Conclusion:**

This NLP algorithm automatically detects the presence of sexual history documentation and its key components in ED and inpatient settings.

## Introduction

Despite the high incidence of sexually transmitted infections (STIs) in the adolescent population, sexual health screenings as recommended by the Centers for Disease Control and Prevention (CDC) and American Academy of Pediatrics (AAP) are not reliably accomplished with well visits alone (1–4). A previous study by Goyal et al. revealed rates of sexual history documentation (SHxD) as low as 21% in the primary care setting and even lower STI screening rates (3). Acute care visits, including hospitalizations and emergency department (ED) encounters, provide additional opportunities to perform sexual health screening and reduce morbidity associated with delayed diagnosis. However, rates of SHxD range from 18 to 70% in the pediatric ED and 43–62% in the inpatient setting, leaving substantial room for improvement (5–8). To increase sexual health screening rates, quality improvement efforts need reliable process measures that can be easily tracked while conducting Plan-Do-Study-Act cycles (9). Automated detection of SHxD would facilitate these studies to address an important gap in the provision of evidence-based care for adolescents.

A substantial amount of data in electronic health records (EHRs) is stored as free text, which provides freedom of expression to clinicians (10, 11). Natural Language Processing (NLP) techniques can extract structured information from free text to identify patients meeting inclusion criteria for studies and inform clinical decision support, among other purposes (12, 13). This technique has been employed to identify different aspects of social history documented within clinical notes (14). To our knowledge, only one prior study has created a machine learning model to detect the presence of social and behavioral determinants of health within clinician notes and specifically looked for sexual history (15). A simpler example of NLP is rule-based algorithms based on regular expressions. Regular expressions are search patterns defined by character types (16). For example, the pattern “sex[a-z,.„/]” matches the word “sex” followed by letters, a period, a space, or a slash. Thus, it matches “sexual activity,” “does not have sex,” and “no sex/drug use,” but does not match “Sex: Male.” While this approach is less sophisticated than more complex machine learning NLP methods, regular expression-based models are comparatively easy to implement and interpret. In the past, researchers have utilized regular expressions to extract information on various topics, such as frequency of tobacco use and housing issues, from free-text with relatively good success (17–20). For example, Turchin et al. published an algorithm that recognized documentation of elevated blood pressure with 98% sensitivity and 93% positive predictive value (PPV) and documentation of anti-hypertensive treatment intensification with 84% sensitivity and 86% PPV (20).

In this study, we aimed to develop and validate a rule-based algorithm using regular expressions to detect SHxD among adolescents who received care in the pediatric ED or inpatient setting.

## Methods

### Design

At a single academic children's health system composed of three urban hospitals, we extracted a retrospective cross-section of pediatric ED provider notes from adolescent visits with specific gastrointestinal or genitourinary/gynecologic chief complaints. Our team consisted of an adolescent medicine physician, a pediatric hospital medicine fellow, a pediatric emergency medicine fellow, a pediatric hospitalist/clinical informaticist, as well as a human factors engineer. As a team, we reviewed 10 charts, or provider notes for the included patient visit, and used them to develop the initial regex. All five authors agreed upon the presence/absence of SHxD, or any mention of sexual activity, and components of the CDC recommended 5P's question framework (Partners, Practices, Past History of STIs, Protection from STIs, and Prevention of Pregnancy), or any plausible response to the suggested questions (21). Since there were no disagreements among all five authors and the task was straightforward, all subsequent notes were manually reviewed for the presence/absence of SHxD and components of the 5Ps by one author—the pediatric emergency medicine fellow or the pediatric hospital medicine fellow depending on their area of clinical practice.

While reviewing the initial charts, we iteratively generated a NLP algorithm based on regular expressions defined by subject matter experts—the clinicians noted above. We then validated this algorithm using a retrospective cross-section of inpatient admission notes for adolescents within the same health system. Our gold standard for the presence of SHxD and components of the 5P's was manual chart review by either a pediatric emergency medicine or a hospital medicine fellow as stated above. For each model, we calculated the following performance measures: sensitivity, specificity, negative predictive value (NPV), positive predictive value (PPV), and F1 score. We chose these measures as they are commonly used to assess screening tools and Ford et al. utilized the same metrics when comparing previously published NLP algorithms (12). The entire process of reviewing the notes, creating the NLP, and calculating its performance measures took ~6 months.

Notes were extracted from the Epic Systems Clarity database using Microsoft SQL server v18.3.1 (Redman, WA) and regular expressions were applied within the same software using Transact-SQL. The institutional review board of the children's health system approved this study.

### Algorithm development

We extracted the full text of ED provider notes from the EHR (Epic systems©) for all ED visits of adolescents 13–21 years old with the following chief complaints: genitourinary complaint, menstrual problem, vaginal problem/discharge/bleeding, penis/scrotum problem, testicular pain, pelvic pain, abdominal cramping, dysuria, urinary complaint, hematuria, polyuria, or anal mass/pain. Since ED visits are typically focused on acute problems, we chose chief complaints that could be caused by a STI. Patients seen for alleged sexual assault were excluded as these notes are made private at our institution. Notes were written by either attending physicians, residents, or nurse practitioners.

As a group, the authors reviewed a sample of 10 notes to identify commonly used phrases (e.g., “denied sexual activity” and “sexually active”) in SHxD and for the 5P's. Based on these phrases, the team developed a set of candidate regular expressions to identify sexual history and 5P documentation in the notes. We then applied these candidate regular expression models to notes from 2 months at a time (range: 136–201 notes) and compared model classification to manual review. We reviewed misclassifications as a team, made adjustments to the regular expressions, and applied them to an additional 2 months' worth of notes. This technique, known as the top-down approach, is an established method for creating regular expressions (16). We made no further modifications to each regular expression model when either (1) the sensitivity and specificity reached >95% on a new sample or (2) the authors could not identify any new patterns from misclassified notes that could be practicably incorporated. Our last cohort consisted of 179 notes. These were manually reviewed by the pediatric emergency medicine fellow alone since agreement on the presence/absence of SHxD and components of the 5P's was already established by the group. The final NLP rules and regular expressions are described in Table 1.

**Table 1:** Regular expressions used in final rule-based natural language processing algorithm.

**Information searched**	**Regular expressions** * **used in query**	**Examples**
Sexual history	sex[a-z,\.,\s,\/]|intercourse	MATCH: “sexual activity,” “does not have sex,” no sex/drug use,” “had sex” NOT A MATCH: “Sex: Male”
Protection used	protect|condom condom cath|child protect	**MATCH:** “uses protection,” “uses condoms some of the time” **NOT A MATCH:** “condom catheter,” “child protection services”
STI testing offered/previously performed	GC[∧S]|G\/C|gonorr| gonnor|gonor|gonoc|chlam| ST[I|D]\s(test|screen|lab)| test(ing|ed|\s)\sfor\sST[I|D] chlam pneumo|chlamydia pneumo	**MATCH:** “will send GC/CT,” “STD testing,” “test for STI,” “chlamydia” **NOT A MATCH:** “chlamydia pneumonia”
Not sexually active	denies (any sex|history of sex|hx of sex|intercourse|hx of intercourse|being sexual|ever being sexual)|no sex|never (had sex|been sexual)|not sexually active	**MATCHES:** “denies ever being sexually active,” “never been sexually active” **NOT A MATCH:** “denies ever having been sexually active”
Partner gender	Within substring of text 50 characters prior to and 50 characters after/ sex[a-z,\.,\s,\/]|intercourse/ male/boy/girl/men/man	**MATCH:** “has sex with female partners,” “hx of sexual activity with women” **NOT A MATCH:** “interested in men” (with no mention of sex or intercourse within 50 characters)
Type of sexual practice	(oral|vaginal|anal)\s(sex|penetration| intercourse)	**MATCH:** “has had oral sex,” “anal penetration” **NOT A MATCH:** “has had sex—both oral and anal”
Contraception used	OCP|contraceptive|contraception, birth control|planon|IUD|nuvaring| depo	**MATCH:** “is on OCP,” “Depo-provera 1 month ago,” “on birth control for PCOS” **NOT A MATCH:** “on mini pill”

* *All regular expressions are given in Python format and were multi-line and case insensitive*.

### Algorithm validation

We identified adolescents, 14–19 years old, who were admitted to general pediatrics services over 2 months (*n* = 321) at the same health system. Ages of these patients were slightly different than in the ED population solely due to the desire to study this population in a future study. There were no restrictions based on chief complaint. We extracted the History & Physical (H&P) notes for each encounter. Notes were written by either attending physicians or residents. The model's reported predictions for the presence of SHxD and the 5P's were compared to manual review performed by the pediatric hospital medicine fellow. The same performance measures were calculated.

## Results

### Emergency department

The model was able to correctly identify the 97 of 179 (54%) notes with a documented sexual history (Table 2). Therefore, the NLP model exhibited 100% sensitivity and specificity for detecting the presence of SHxD (Table 3). It also had perfect accuracy for detection of type of sexual practice and contraception use. Protection used and STI testing offered/previously performed were identified with high sensitivity (100% and 98.2%, respectively) and specificity (97.9% and 98.4%, respectively). Documentation of a patient as not sexually active was detected with high specificity (100%) but lower sensitivity (89.7%). Documentation of partner gender had high sensitivity (100%) but lower specificity (77.6%).

**Table 2:** Frequency of sexual history and the 5P's in notes based on manual review.

**Category**	**ED notes (*****n*** **= 179)**	**Inpatient notes (*****n*** **= 321)**
Sexual history	97/179 (54.2%)	97/321 (30.2%)
Protection used	33/179 (18.4%)	23/321 (10.0%)
STI testing offered/previously performed	56/179 (31.3%)	37/321 (11.5%)
Not sexually active	39/179 (21.8%)	52/321 (16.2%)
Partner gender	26/179 (14.5%)	36/321 (11.2%)
Type of sexual practice	9/179 (5.0%)	8/321 (2.5%)
Contraception used	32/179 (17.9%)	21/321 (6.5%)

**Table 3:** Performance measures of the rule-based natural language processing algorithm in the ED and inpatient settings.

**Information searched**	**Performance measures for ED test group**					**Performance measures for inpatient validation group**				
	**Sensitivity**	**Specificity**	**PPV** *	**NPV** *	**F1 score**	**Sensitivity**	**Specificity**	**PPV** *	**NPV** *	**F1 score**
Sexual history	97/97 (100.0%)	82/82 (100.0%)	97/97 (100.0%)	82/82 (100.0%)	100.0%	97/97 (100.0%)	220/224 (98.2%)	97/101 (96.0%)	220/220 (100.0%)	98.0%
Protection used	33/33 (100.0%)	143/146 (97.9%)	33/36 (91.7%)	143/143 (100.0%)	95.7%	23/23 (100.0%)	290/298 (97.3%)	23/31 (74.2%)	290/290 (100.0%)	85.2%
STI testing offered/previously performed	55/56 (98.2%)	121/123 (98.4%)	55/57 (96.5%)	121/122 (99.2%)	97.3%	23/37 (85.2%)	268/294 (91.2%)	23/49 (46.9%)	268/282 (95.0%)	60.5%
Not sexually active	35/39 (89.7%)	126/126 (100.0%)	35/35 (100.0%)	126/130 (96.9%)	94.6%	41/52 (78.8%)	268/269 (99.6%)	41/42 (97.6%)	268/279 (96.1%)	87.2%
Partner gender	26/26 (100.0%)	118/152 (77.6%)	26/60 (43.3%)	118/118 (100.0%)	60.4%	33/36 (91.7%)	220/285 (77.2%)	33/98 (33.7%)	220/223 (98.7%)	49.3%
Type of sexual practice	9/9 (100.0%)	170/170 (100.0%)	9/9 (100.0%)	170/170 (100.0%)	100.0%	7/8 (87.5%)	313/313 (100.0%)	7/7 (100.0%)	313/314 (99.7%)	93.3%
Contraception Used	32/32 (100.0%)	146/146 (100.0%)	32/32 (100.0%)	146/146 (100.0%)	100.0%	21/21 (100.0%)	295/300 (98.3%)	21/26 (80.8%)	295/295 (100.0%)	89.4%

* *PPV, positive predictive value; NPV, negative predictive value*.

### Inpatient

Out of the 321 H&P notes included in the inpatient cohort, 97 (30%) included a sexual history (Table 2). The NLP model demonstrated 100% sensitivity and 98.2% specificity for detecting the presence of a SHxD (Table 3). It had excellent performance in detection of protection used (sensitivity 100%, specificity 97.3%) and contraception use (sensitivity 100%, specificity 98.3%). The model detected denial of sexual activity and type of sexual activity with high specificity (99.6 and 100%, respectively), but lower sensitivity (78.8 and 87.5%, respectively). Model performance was lower for detection of STI testing offered/previously performed as well as for partner gender.

## Discussion

To our knowledge, this is the first study to develop and validate an automated method using regular expressions to extract information regarding documentation of sexual history in the adolescent population that can be applied at scale. Prior studies reporting the rate of SHxD in the pediatric ED and inpatient setting have relied on manual chart review. This approach can be cumbersome and limit the number of encounters included in these studies. The largest known study to date in the inpatient setting reported the presence and quality of SHxD for 752 adolescents (5). We employed this algorithm to a cohort of 1,987 ED patients (22). The methods described in this study could also easily be applied to all adolescent encounters in a health system and track changes in SHxD over time.

Although there are no rule-based algorithms for identifying SHxD in any age group, a machine learning model was described by Feller et al. to detect documentation of social and behavioral determinants of health in adults, including sexual history (15). Inputs for their model consisted of both free-text from clinical notes as well as structured EHR data (diagnoses, procedures, laboratory tests, and demographics). They compared the performance of their model for detecting SHxD based on free-text alone vs. free-text plus structured EHR data. Both models had a reported F1 score of 79%, which was lower than that of our algorithm. Previously published, rule-based algorithms have been employed to extract other information from free text or identify specific patient populations, such as patients with acute respiratory infection, obesity and associated comorbidities, and a history of tobacco use (23–25). Ford et al. performed a systematic review of these algorithms and calculated their average performance measures to be the following: sensitivity of 96%, PPV of 85%, and F1 score of 49% (12). Our rule-based algorithm demonstrated a sensitivity, PPV, and F1 score greater than this for detecting sexual history.

For recognizing the 5P's, our algorithm did not perform at similar high standards for a few components. The lowest sensitivity was 78.8% for identifying documentation regarding not sexually active. The lowest F1 scores were for detecting partner gender (49%) and STI testing offered/previously performed (61%). Many false positives for partner gender occurred if “male” or “female” were noted in response to a different question, such as gender identity. The use of abbreviations, such as “BF” for boyfriend, lead to a few false negatives. As for STI testing, many false positives occurred due to the use of “g/c” text in other context, such as “no clubbing/cyanosis.” Further iterations could be performed to address these false positives. However, even the current lowest performance measures would be sufficient for quality improvement initiatives. In addition, regular expression-based models are comparatively easier to implement and require fewer technical resources than more complex machine learning methods that may allow for higher sensitivity and specificity.

In general, our model demonstrated better performance measures in the ED setting compared to the inpatient setting. Since asking a sexual history was pertinent to the chief complaints selected for the ED encounter notes, the rate of SHxD and documentation of the 5P's were generally higher in the ED notes compared to the inpatient notes. Physicians in the inpatient setting may not have elected to ask a sexual history if the chief complaint was not related to STIs. Lower inpatient SHxD and 5P documentation rates likely contributed to lower PPVs for the model when applied in the inpatient setting. Therefore, it is likely that the PPV may vary based on the baseline documentation rates of the healthcare setting in which it is employed. Though, this should not affect the sensitivity or specificity of the tool, which was high in both settings.

This study is limited in that the model development and validation were performed at a single institution. Therefore, performance of the algorithm may vary when implemented at other institutions due to differences in documentation practices, such as distinct note templates and abbreviations. Although this method has not been validated at other centers, the regular expressions are not specific to an HER vendor and could be used in multicenter studies. Another limitation was the need for repeated manual chart review to improve performance of the algorithm. Some manual chart review will likely be necessary when validating the algorithm at a new institution as well. However, once complete, the algorithm can be applied to numerous charts, avoiding grueling manual review of individual charts to perform a large study.

## Conclusion

Within this study, the SHxD rate in the ED was 54% despite limiting notes to those for encounters with a related chief complaint. The SHxD was even lower in the inpatient setting. In order to reduce the morbidity from preventable and treatable STIs in adolescents, there is a strong need for initiatives to improve screening practices in acute care settings and, therefore, enhance accessibility. The rule-based algorithm developed and validated in this study can be used at other institutions to facilitate these research and quality efforts. In addition, as health systems work to protect adolescent confidentiality during the movement to Open Notes, this method could be used to determine if sexual histories are documented less frequently by surveying both shared and confidential notes (26). Future studies could develop NLP models for other core adolescent health services, such as mental health screening, and advance quality improvement efforts for a variety of adolescent health issues.

## Data Availability Statement

The data analyzed in this study is subject to the following licenses/restrictions: dataset details private patient notes protected according to HIPAA and our IRB policy. Requests to access these datasets should be directed to gmukher@emory.edu.

## Author contributions

CR contributed to the design of the algorithm, performed analysis for emergency department charts, reviewed the manuscript, and approved the final version as submitted. GM performed the analysis for inpatient charts, drafted the initial manuscript, revised the manuscript, and approved the final version as submitted. HG helped design the algorithm, reviewed and revised the manuscript, and approved the final manuscript as submitted. SK and EO created the algorithm, reviewed and revised the manuscript, and approved the final version as submitted. All authors contributed to the article and approved the submitted version.

## Conflict of interest

EO is a co-founder and has equity in Phrase Health, a clinical decision support analytics company. He receives no direct revenue. EO is also a PI on a Phase 1 and Phase 2 STTR grant with Phrase Health from the National Library of Medicine and the National Center for Advancing Translational Science. He receives salary support through these grants. The remaining authors declare that the research was conducted in the absence of any commercial or financial relationships that could be construed as a potential conflict of interest.

## Publisher's note

All claims expressed in this article are solely those of the authors and do not necessarily represent those of their affiliated organizations, or those of the publisher, the editors and the reviewers. Any product that may be evaluated in this article, or claim that may be made by its manufacturer, is not guaranteed or endorsed by the publisher.
